# Impact of the COVID-19 Pandemic on Odontogenic Abscess Clinical Patterns and Predictive Factors: A Retrospective Cross-Sectional Study

**DOI:** 10.3390/jcm14196953

**Published:** 2025-10-01

**Authors:** Kacper Nijakowski, Stanisław Ksel, Olesya Marushko, Aleksy Nowak, Jakub Jankowski, Jacek Kwiatkowski, Olena Marushko, Łukasz Słowik, Maciej Okła

**Affiliations:** 1 Department of Conservative Dentistry and Endodontics, Poznan University of Medical Sciences, 60-812 Poznan, Poland; jjankowski41@wp.pl; 2 The Student Scientific Society, Poznan University of Medical Sciences, 60-806 Poznan, Poland; 3 Department of Maxillofacial Surgery, Poznan University of Medical Sciences, 60-355 Poznan, Poland; nowak.aleksy@usk.poznan.pl (A.N.); lukasz.slowik@ump.edu.pl (Ł.S.); 4 Ludwik Rydygier Collegium Medicum, Nicolaus Copernicus University in Toruń, 85-067 Bydgoszcz, Poland

**Keywords:** COVID-19, odontogenic abscess, hospitalisation, procalcitonin, antibiotic therapy

## Abstract

**Background/Objectives**: The COVID-19 pandemic disrupted healthcare systems globally, with dental services significantly limited due to infection control measures. This study investigates the impact of the pandemic on the clinical presentation, management, and outcomes of odontogenic abscesses over three distinct periods. **Methods**: A retrospective study was conducted at University Clinical Hospital (Poznan, Poland), which included adult patients hospitalised for odontogenic infections between March 2019 and February 2022. The cohort comprised 101 patients (median age: 33 years; 59.41% male), with admissions distributed across pre-pandemic (37.62%), pandemic (19.80%), and post-pandemic (42.57%) periods. Clinical, biochemical, and radiographic data were analysed. **Results**: No statistically significant differences were found between periods for abscess severity, hospitalisation length, or inflammatory marker levels. Elevated procalcitonin (Rs = 0.289, *p* = 0.005), C-reactive protein (Rs = 0.385, *p* < 0.001), and body mass index (Rs = 0.253, *p* = 0.011) independently predicted longer hospital stays. In regression modelling, procalcitonin (β = 0.464, *p* = 0.001) and prior outpatient antibiotic use (β = 0.281, *p* = 0.038) were mainly associated with larger abscess volumes, while comorbidities (β = 0.262, *p* = 0.025), longer hospitalisation (β = 0.594, *p* = 0.001) and abscess volume (β = −0.294, *p* = 0.040) increased the risk of reoperation. **Conclusions**: The study highlights clinically important findings linked to delayed dental care and increased systemic inflammation related to the pandemic. Elevated procalcitonin and CRP levels provide prognostic information that can guide early triage, risk stratification, and decisions regarding surgical versus outpatient management. These findings emphasise the importance of maintaining essential dental services, implementing preventive strategies, and optimising management protocols to reduce the risk of severe infections and improve patient outcomes during healthcare disruptions.

## 1. Introduction

Odontogenic infections represent a significant global health burden, frequently necessitating urgent hospital intervention due to their potential for rapid progression and life-threatening complications. Although most odontogenic infections can be managed effectively in outpatient settings with timely dental care, delays in treatment can lead to severe morbidity, including spread to deep fascial spaces, systemic infection, and increased need for invasive surgical procedures [1,2]. The COVID-19 pandemic, declared in March 2020, dramatically disrupted healthcare systems worldwide, with dental services among the most severely affected. Dental practitioners faced heightened risks of SARS-CoV-2 transmission due to aerosol-generating procedures and prolonged patient proximity [3]. In Poland, as elsewhere, access to routine dental care was curtailed by lockdowns and infection control protocols, with services restricted primarily to emergency cases [4,5,6].

Emerging evidence suggests that pandemic-related restrictions disrupted access to routine and emergency dental care, exacerbating untreated dental disease and potentially increasing the incidence and severity of odontogenic infections [7]. Preliminary studies from various regions have reported a higher frequency and severity of odontogenic abscesses during the pandemic [8,9,10], but most were limited to single centres or short-term observations. Importantly, few investigations have examined whether increased infection severity coincided with changes in clinical management protocols, etiological factors, or patient outcomes across distinct pandemic phases. It remains unclear to what extent disruptions in dental care, altered patient behaviour, or broader systemic factors contributed to these observed changes. A clearer understanding of these dynamics is essential for identifying modifiable risk factors, optimising emergency management, and strengthening healthcare preparedness for future crises.

The primary objective of this study was to assess the clinical features and management of odontogenic abscesses across the pre-pandemic, pandemic, and post-pandemic periods, testing the hypothesis that the COVID-19 pandemic influenced abscess presentation, severity, and treatment. Secondary objectives included exploring associations between local etiological factors and time periods, as well as identifying clinical and biochemical predictors of hospitalisation length, abscess size, and risk of reoperation. These findings provide critical insights into the pandemic’s effects on odontogenic infection outcomes and highlight prognostic factors relevant for clinical decision-making.

## 2. Materials and Methods

### 2.1. Study Design and Data Collection

This retrospective cohort study analysed medical records of patients hospitalised with odontogenic infections at University Clinical Hospital (Poznan, Poland) between March 2019 and February 2022, divided into three distinct periods: pre-pandemic (March 2019–February 2020), pandemic (March 2020–February 2021), and post-pandemic (March 2021–February 2022). The terms were defined conventionally with reference to the phase of the pandemic’s greatest intensity, without corresponding to an epidemiological classification.

Inclusion criteria included patients aged ≥ 18 years hospitalised with a confirmed diagnosis of odontogenic infection based on clinical examination and radiographic findings. Exclusion criteria included patients without the need of hospitalisation, with non-odontogenic maxillofacial infections, with immunocompromised status unrelated to dental origin, and with heavily incomplete medical records.

Collected variables included age, gender, location, body mass index (BMI), hospitalisation length, symptoms, comorbidities, use of stimulants, associated tooth/teeth, affected anatomical areas, abscess volume, type of anaesthesia, administered antibiotics, leukocyte levels, C-reactive protein (CRP) levels, and procalcitonin levels, as well as the need to reoperate. Assessment of abscess volume was performed using CT segmentation, conducted by a radiology specialist with over a decade of professional experience. The database was stored using the Apple Inc. Numbers version 11.2 spreadsheet.

This study was designed and reported in compliance with the STROBE statement for cross-sectional research.

### 2.2. Statistical Analysis

The group characteristics were presented using medians and interquartile ranges (IQR) for continuous variables (due to non-compliance with the normal distribution as determined by the Shapiro–Wilk test) and using counts and percentages for categorical variables. Quantitative variables were compared across time periods (before, during, and after the pandemic) using the Kruskal–Wallis test.

Additionally, for categorical variables such as the period relative to the pandemic and local factors in the oral cavity (side—maxilla/mandible, quadrants, and type of causative tooth), a multidimensional correspondence analysis (MCA) was conducted. Based on the scree plot analysis, a two-dimensional model was selected, in which the strongest relationships are between the points forming the sharpest angles (with the vertex at the origin of the coordinate system).

Moreover, Spearman correlation analysis was performed between length of hospitalisation, abscess volume, and number of affected spaces and other demographic and clinical parameters. Similarly, an attempt was made to construct multiple regression models for length of hospitalisation, abscess volume, number of affected spaces, and the need for reoperation using the stepwise forward selection method.

Missing data were handled using the imputation method, and outliers were assessed visually and statistically; extreme values were retained/excluded as appropriate for each analysis. For all analyses, a significance level of alpha = 0.05 was assumed. The analyses were conducted using Statistica version 13.3 (StatSoft, Cracow, Poland).

## 3. Results

### 3.1. Characteristics of the Study Group

The study group consisted of 101 patients with a median age of 33 years (IQR: 26–47)—Table 1. The majority were male (59.41%), and the median BMI was 24.39 kg/m^2^ (IQR: 21.36–27.41). Most participants resided in urban areas (69.31%), while 30.69% were from rural regions. With respect to the time of admission, 37.62% of patients were treated in the pre-pandemic period, 19.80% during the pandemic, and 42.57% in the post-pandemic phase. Comorbidities were present in 31.68% of individuals, with the most frequent being hypertension (15.84%), followed by cardiovascular diseases (9.90%), diabetes (5.94%), hypothyroidism (4.95%), respiratory conditions (3.96%), mental health disorders (3.96%), and haematologic diseases (1.98%). The use of stimulants was reported by 37.62% of patients. Tobacco use was the most prevalent (35.64%), while alcohol and drug use were reported by 4.95% and 0.99% of individuals, respectively.

Detailed clinical characteristics are presented in Table 2. The median length of hospitalisation was 5 days (IQR: 4–6), and the median abscess volume was 18.01 cm^3^ (IQR: 6.63–42.77). The number of affected anatomical spaces had a median value of 1 (IQR: 1–2).

Upon admission, laboratory tests revealed elevated inflammatory markers, with a median white blood cell count (WBC) of 14.25 × 10^3^/μL (IQR: 11.60–17.03), a CRP concentration of 138.3 mg/L (IQR: 82.5–210.7), and a procalcitonin of 0.13 ng/mL (IQR: 0.08–0.38). The most frequently reported symptoms included pain (93.07%), swelling (82.18%), trismus (60.40%), and dysphagia (38.61%). Fever was present in 21.78% of cases, dyspnoea in 6.93%, and headache in 3.96%. The most commonly involved anatomical regions were the submandibular space (66.34%), followed by the submental (11.88%), cervical (10.89%), pterygomandibular (8.91%), and submasseteric (8.91%) areas. Less frequently involved spaces included the mouth floor (5.94%) and canine fossa (4.95%). Rare locations, such as the orbit, pterygopalatine and sublingual spaces, were not affected in this cohort. Phlegmon was observed in 24.75% of patients and empyema in 0.99%.

Most infections were localised to the mandible (80.20%), with only 6.93% affecting the maxilla and 12.87% being bilateral or indeterminate. The most common causative teeth were third molars (47.52%) and other molars (29.70%), with fewer infections originating from premolars (4.95%), canines (0.99%), and incisors (1.98%).

General anaesthesia was used in 85.15% of surgical interventions, and reoperation was required in 5.94% of cases. Half of the patients (50.50%) received antibiotic therapy prior to hospitalisation, most commonly clindamycin (30.69%), followed by amoxicillin with clavulanic acid (9.90%) and metronidazole (5.94%). During hospitalisation, 97.03% of patients received antibiotics, primarily metronidazole (68.32%), amoxicillin with clavulanic acid (31.68%), clindamycin (24.75%), cefuroxime (19.80%), and ceftriaxone (16.83%). Hospital antibiotic therapy was most often combined with several types of drugs and less frequently administered sequentially.

### 3.2. Manifestations of Odontogenic Abscesses Across Periods Relative to the COVID-19 Pandemic

Table 3 presents a comparison of selected clinical and laboratory parameters among patients admitted in the pre-pandemic, pandemic, and post-pandemic periods. No statistically significant differences were observed for any of the variables analysed.

Median age tended to be higher in the pre-pandemic group (39 years; IQR: 30–52) compared to the pandemic (33.5 years; IQR: 23–39.5) and post-pandemic groups (30 years; IQR: 25–45), although this difference was not statistically significant (*p* = 0.265). Similarly, no differences were observed in BMI (*p* = 0.723), hospitalisation length (*p* = 0.883), abscess volume (*p* = 0.857), or the number of affected anatomical spaces (*p* = 0.982).

Laboratory parameters at admission, including WBC count, CRP, and procalcitonin levels, also showed no significant variation between groups (*p* = 0.543, *p* = 0.863, and *p* = 0.176, respectively). Notably, while procalcitonin levels appeared elevated during the pandemic period (median: 0.17 mg/mL; IQR: 0.09–1.06) compared to the pre- and post-pandemic phases, this trend did not reach statistical significance.

**Figure 1:**
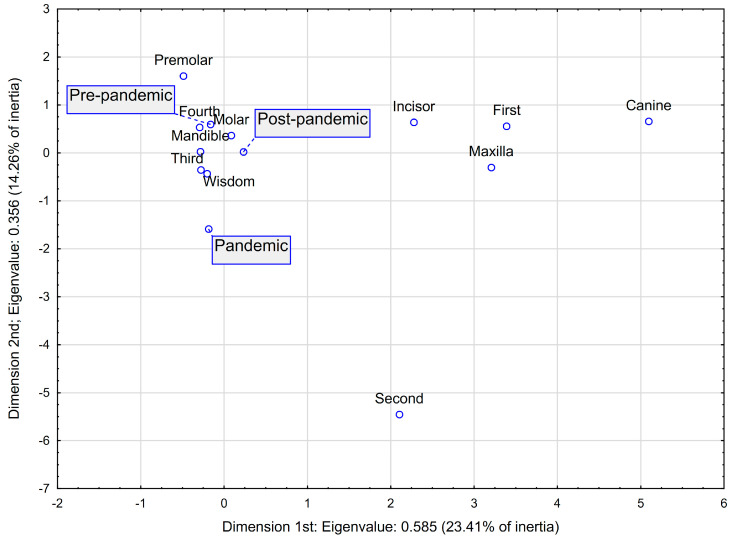
Multidimensional correspondence analysis on the impact of pandemic periods on the most common reasons for odontogenic abscesses: two-dimensional plot. Interpretation: The plot displays the relationships between different categorical variables. Points that are located closer to one another suggest an association, meaning they are more likely to occur together within the study population. The strength of the relationship can additionally be determined by assessing the angle between the variables under study (with the vertex at the origin of the coordinate system, 0,0)—the smaller the angle, the stronger the relationship.

Figure 1 presents the results of a two-dimensional model derived from MCA, exploring the associations between periods relative to the COVID-19 pandemic (pre-pandemic, pandemic, and post-pandemic) and local etiological factors of odontogenic abscesses, including the location (maxilla vs. mandible and quadrants) and type of the causative tooth (e.g., incisor, molar, and wisdom tooth).

The pre-pandemic period clusters near the mandibular location (including the third and the fourth quadrants) with premolars, suggesting these locations and tooth types were more frequently associated with abscesses before the pandemic. The post-pandemic period is positioned close to molars, indicating a shift toward more advanced odontogenic infections after the pandemic.

The pandemic period is notably distant from most local factors, possibly reflecting a reduction in typical presentations or delayed care-seeking behaviour due to restricted dental service availability during that time. Conversely, maxillary abscesses and infections originating from canines, incisors, and the first quadrant appear more isolated, suggesting a weaker overall association with any specific pandemic period.

The dispersion of categories illustrates variation in etiological patterns that may correspond with changes in patient behaviour and healthcare system accessibility during the pandemic phases. Detailed parameters of MCA are presented in Table 4.

### 3.3. Prognostic and Predictive Factors for Odontogenic Abscesses and Their Treatment

Spearman’s rank correlation analysis was conducted to assess the relationships between hospitalisation length, abscess volume, and the number of affected anatomical spaces and various demographic and clinical variables (Table 5).

A statistically significant positive correlation was found between hospitalisation length and several parameters: BMI (Rs = 0.253; *p* = 0.011), CRP levels at admission (Rs = 0.385; *p* < 0.001), and procalcitonin concentration (Rs = 0.289; *p* = 0.005). Additionally, longer hospitalisation was significantly associated with lower likelihood of in-hospital antibiotic administration (Rs = −0.222; *p* = 0.026). Although a positive correlation with comorbidities was observed (Rs = 0.195; *p* = 0.051), this result approached but did not reach statistical significance.

Abscess volume showed a significant positive correlation only with WBC count on admission (Rs = 0.342; *p* = 0.018), with no statistically significant associations found with age, BMI, and inflammatory markers such as CRP or procalcitonin.

The number of affected anatomical spaces correlated significantly with comorbidities (Rs = 0.256; *p* = 0.010), WBC count (Rs = 0.301; *p* = 0.002), CRP (Rs = 0.393; *p* < 0.001), and procalcitonin levels (Rs = 0.258; *p* = 0.013). A strong positive correlation was also observed between affected area number and hospitalisation length (Rs = 0.495; *p* < 0.001), suggesting that more extensive infections may contribute to prolonged inpatient care.

Multiple regression modelling was conducted to identify predictors of clinical outcomes, including hospitalisation length, abscess volume, the number of affected anatomical spaces, and the need for reoperation (Table 6). The model for hospitalisation length demonstrated excellent explanatory power (R^2^ = 0.815, *p* < 0.001). Significant positive predictors included admission procalcitonin concentration (β = 0.315, *p* = 0.003), CRP level (β = 0.240, *p* = 0.010), abscess volume (β = 0.308, *p* = 0.002), and reoperation (β = 0.299, *p* = 0.003). There was a borderline-negative association with stimulant use (β = –0.154, *p* = 0.054), while prior antibiotic therapy did not significantly influence hospitalisation duration.

The abscess volume model yielded a moderate fit (R^2^ = 0.480, *p* = 0.001). Higher procalcitonin levels (β = 0.464, *p* = 0.001) and prior outpatient antibiotic treatment (β = 0.281, *p* = 0.038) were positively associated with abscess size. Interestingly, presentation in the post-pandemic period was associated with significantly smaller abscesses (β = –0.274, *p* = 0.039).

For the number of affected anatomical areas, the model explained a smaller proportion of the variance (R^2^ = 0.283, *p* = 0.023), with WBC count at admission emerging as the only significant predictor (β = 0.351, *p* = 0.019). Living in an urban area (β = 0.252, *p* = 0.088) showed a trend toward significance.

The multiple regression model for reoperation demonstrated good explanatory value (R^2^ = 0.603, *p* < 0.001). Longer hospitalisation (β = 0.594, *p* = 0.001) and the presence of comorbidities (β = 0.262, *p* = 0.025) were independently associated with the need for reoperation. Interestingly, larger abscess volumes were inversely related to reoperation risk (β = –0.294, *p* = 0.040), while elevated procalcitonin levels showed a non-significant trend (β = 0.301, *p* = 0.074). However, it should be emphasised that there were only six reoperation cases during the study period.

## 4. Discussion

The COVID-19 pandemic precipitated unprecedented disruptions in global healthcare systems, with dental services disproportionately affected due to the aerosol-generating nature of procedures and heightened transmission risks [11]. This retrospective study examines whether these disruptions influenced the clinical presentation, severity, and management of odontogenic abscesses across pre-pandemic, pandemic, and post-pandemic periods. Although no statistically significant differences were observed in hospitalisation duration or abscess severity, several clinically relevant trends emerged, corroborating and expanding upon existing literature.

Multidimensional correspondence analysis (MCA) revealed a post-pandemic trend toward infections associated with mandibular third molars, suggesting that delayed dental care during lockdowns may have contributed to the progression of initially manageable conditions into abscesses necessitating hospitalisation. This finding aligns with international studies reporting increased complications from untreated dental disease during the pandemic. For instance, a UK-based study documented a rise in hospital admissions for mandibular dental abscesses in 2020 compared to 2019, alongside a marginal increase in hospital stay duration [12]. Similarly, a single-centre study in Greece reported more severe maxillofacial infections post-lockdown, with an increased proportion of patients requiring intensive care and prolonged hospitalisation [13].

The relative isolation of pandemic-period cases from typical etiological patterns in our MCA may reflect the well-documented decline in dental attendance during this time [14]. Global surveys indicate widespread dental service disruptions, with emergency cases prioritised. In Poland—where this study was conducted—dental clinics operated at reduced capacity during peak restrictions, potentially explaining the smaller proportion of pandemic-era cases (19.8%) in our cohort [15].

Our findings demonstrate that inflammatory markers (CRP, procalcitonin, and WBC) strongly predicted hospitalisation duration, consistent with prior research validating their utility in assessing odontogenic infection severity. Meta-analyses have established CRP > 100 mg/L as predictive of complicated odontogenic infections [16]. The robust association with procalcitonin (β = 0.315, *p* = 0.003) supports emerging evidence of its superiority over CRP in distinguishing bacterial infections, particularly in maxillofacial contexts [17]. Notably, the median CRP level observed (138.3 mg/L) exceeds pre-pandemic benchmarks, potentially indicating more advanced presentations during the study period. However, direct comparisons are confounded by heterogeneity in measurement timing and patient populations. Studies suggest that elevated CRP during lockdowns may reflect delayed hospital attendance, with patients resorting to analgesics before seeking care [12].

High rates of pre-hospital antibiotic use (50.5%)—primarily clindamycin (30.69%)—reflect global trends in dental prescribing during the pandemic, when delayed definitive treatment led to increased reliance on empiric therapy. Regression analysis revealed that prior antibiotic use was associated with larger abscess volumes (β = 0.281, *p* = 0.038), suggesting that inappropriate antibiotic regimens may suppress symptoms without effectively resolving infections. This aligns with broader findings that 97.03% of hospitalised patients received antibiotics, consistent with worldwide increases in broad-spectrum antibiotic use due to their perceived efficacy and oral bioavailability amid restricted dental access [18]. Notably, clindamycin (30.69%) and amoxicillin–clavulanate (9.90%) were the most frequently prescribed pre-hospital antibiotics in this cohort. However, emerging evidence highlights the risks of clindamycin overuse, including antibiotic resistance and secondary infections such as Clostridioides difficile [19]. These findings underscore the urgent need for improved antibiotic stewardship in dentistry, particularly during public health crises. Ensuring timely access to definitive dental care, alongside evidence-based antibiotic protocols, is critical to mitigating long-term antimicrobial resistance and improving patient outcomes [18].

The predominance of surgical drainage under general anaesthesia (85.15%) contrasts with some European centres—such as centres guided by the European Society of Endodontology, which support conservative management using local operative treatment (e.g., root canal therapy or extraction) without systemic antibiotics in select cases—but aligns with Polish guidelines emphasising definitive intervention for odontogenic infections [20]. The reoperation rate in our cohort (5.94%) compares favourably to pre-pandemic reports (8–12%). However, the significant association between comorbidities and reoperation risk (β = 0.262, *p* = 0.025) underscores the vulnerability of medically complex patients, consistent with findings that diabetes increases treatment failure odds by 3.1-fold. The urban predominance (69.31%) in our cohort may reflect healthcare access disparities, as rural Polish populations have 30% fewer dentists per capita, highlighting systemic inequities [21].

The study identified BMI as a significant predictor of prolonged hospitalisation (β = 0.253, *p* = 0.011), a finding consistent with emerging evidence linking obesity to worse outcomes in odontogenic infections. Elevated BMI is associated with chronic low-grade inflammation, characterised by increased CRP levels, which may exacerbate infection severity and delay recovery [22]. During the pandemic, sedentary lifestyles and reduced physical activity likely contributed to weight gain, further elevating systemic inflammation and predisposing individuals to infections. Widespread SARS-CoV-2 infection has again highlighted the role of obesity, whose global prevalence increased up to 13%, as a risk factor for both susceptibility to infections and the occurrence of a more severe disease course [22]. Considering the increasing rate of obesity worldwide, it is necessary to investigate possible mechanisms and processes that underlie this association to improve preventive and therapeutic strategies [23]. This aligns with our observations of elevated CRP (median: 138.3 mg/L) and procalcitonin levels, particularly in patients with comorbidities. The interplay between obesity, inflammation, and delayed dental care underscores the need for integrated management strategies addressing both oral and systemic health during public health crises.

Delayed treatment is a well-documented contributor to the increased severity of post-pandemic dental abscesses. While this study did not examine the specific psychological or behavioural factors behind treatment delays, existing research highlights significant barriers to care during the COVID-19 pandemic. Dental anxiety and fears of SARS-CoV-2 transmission emerged as major deterrents, with one study reporting that 73% of patients avoided dental care due to perceived infection risks [24,25]. Economic pressures further exacerbated these delays, particularly as healthcare costs rose disproportionately during lockdowns. In Poland, household healthcare expenditures increased by 9.9% (3.3 billion PLN) between 2021 and 2022, potentially limiting access to timely dental treatment [21]. This trend aligns with broader findings: a survey of 403 patients revealed that 60.8% postponed dental visits due to pandemic-related concerns, with 56.1% specifically citing fears of COVID-19 exposure in clinical settings [26]. Notably, the most common reasons for eventual hospital visits were severe conditions—toothache, abscesses, and impacted teeth (27.8%)—underscoring the consequences of deferred care. These findings emphasise the need for targeted public health strategies to address both psychological barriers (e.g., patient education on clinic safety protocols) and systemic inequities (e.g., affordability measures) during future health crises. Proactive interventions could mitigate treatment delays and reduce the burden of advanced odontogenic infections.

While providing valuable insights into odontogenic abscess patterns during the COVID-19 pandemic, this study has several significant limitations that must be acknowledged. First, as a retrospective single-centre analysis conducted at a tertiary care hospital in Poland, our findings may not fully represent the diverse spectrum of dental care access and treatment protocols across different healthcare systems. The inherent constraints of retrospective data collection limited our ability to standardise all clinical measurements and account entirely for confounding variables such as the exact timing of symptom onset, patient self-treatment behaviours, or detailed socioeconomic factors influencing care-seeking delays.

The sample size of 101 patients, while adequate for initial observations, represents a moderate cohort that may have constrained our statistical power to detect more subtle differences between pandemic periods. This is particularly relevant given the unequal distribution of cases across the three time periods (pre-pandemic: 37.62%, pandemic: 19.80%, and post-pandemic: 42.57%), which, while reflecting real-world healthcare utilisation patterns during the pandemic, may have affected our ability to identify significant temporal trends in abscess severity.

Our reliance on hospital records also introduced potential limitations in data completeness, particularly regarding the documentation of antibiotic regimens prior to hospitalisation and precise timing of symptom progression. The study design did not permit evaluation of significant psychological and behavioural factors that influenced patients’ decisions to delay treatment, such as dental anxiety or specific concerns about COVID-19 transmission in clinical settings—factors shown in other studies to impact care-seeking behaviour during the pandemic significantly.

Despite these limitations, the study makes several significant contributions. The robust associations we identified between inflammatory biomarkers (CRP and procalcitonin) and clinical outcomes provide compelling evidence supporting their use in risk stratification, with procalcitonin showing particular promise as a predictor of hospitalisation duration (β = 0.315, *p* = 0.003). Procalcitonin may serve as an early biomarker for identifying patients at risk of severe odontogenic infections, guiding triage decisions, and informing the urgency of surgical intervention. Its integration into clinical protocols could complement traditional inflammatory markers such as CRP and WBC, particularly in distinguishing bacterial progression from less severe cases, thereby optimising resource allocation and patient outcomes during periods of constrained healthcare access.

These findings have immediate clinical relevance. The significant association between pre-hospital antibiotic use (50.5% of cases, predominantly clindamycin) and larger abscess volumes (β = 0.281, *p* = 0.038) highlights the potential consequences of antibiotic-first strategies when definitive dental care is unavailable. Similarly, the increased reoperation risk among patients with comorbidities (β = 0.262, *p* = 0.025) underscores the particular vulnerability of medically complex patients during healthcare disruptions.

For future research, our results suggest several important directions:

1. Multi-centre prospective studies with standardised data collection protocols;
2. Investigations incorporating patient-reported outcomes and behavioural factors;
3. Longer-term follow-up to assess post-pandemic recovery of dental care systems;
4. Economic analyses of the cost implications of delayed odontogenic infection treatment.

From a public health perspective, these findings strongly support the need to maintain accessible emergency dental services during future healthcare crises while implementing evidence-based antibiotic stewardship programmes. The study provides unique Poland-specific data that complements growing international evidence about the oral health impacts of pandemic-related care disruptions, offering valuable insights for healthcare policymakers and clinicians alike.

## 5. Conclusions

Although no statistically significant differences were observed between the pre-pandemic, pandemic, and post-pandemic groups, our data indicate a post-pandemic trend toward more severe odontogenic infections, particularly those arising from mandibular molars. Elevated procalcitonin and C-reactive protein levels at admission were independent predictors of longer hospitalisation and larger abscess volumes, while the number of affected anatomical spaces was significantly associated with white blood cell count, comorbidities, and inflammatory markers. Longer hospital stays and the presence of comorbidities increased the likelihood of reoperation. Multidimensional correspondence analysis further revealed temporal shifts in abscess aetiology and location, likely reflecting delayed care-seeking and reduced dental service availability during the pandemic.

These findings provide actionable insights for clinical practice. Specifically, inflammatory biomarkers and patient history can guide early triage, inform decisions regarding the timing of surgical drainage versus outpatient management, and support risk stratification for severe odontogenic infections. Under constrained service conditions, our results highlight the importance of early referral thresholds, tailored antibiotic pathways, and preventive dental care to reduce the risk of complications. By linking quantitative findings to practical management strategies, this study offers evidence-based guidance for optimising care during periods of healthcare disruption.

## Figures and Tables

**Table 1:** Demographic characteristics of included patients.

	Median/*n*	IQR/%
Age	33	26–47
Gender:		
Female	41	40.59
Male	60	59.41
BMI	24.39	21.36–27.41
Location:		
Country	31	30.69
City	70	69.31
Period:		
Pre-pandemic	38	37.62
Pandemic	20	19.8
Post-pandemic	43	42.57
Comorbidities:	32	31.68
Diabetes	6	5.94
Hypertension	16	15.84
Hypothyroidism	5	4.95
Respiratory	4	3.96
Cardiovascular	10	9.9
Haematologic	2	1.98
Mental	4	3.96
Stimulants:	38	37.62
Alcohol	5	4.95
Drugs	1	0.99
Tobacco	36	35.64

**Table 2:** Clinical and laboratory characteristics of included patients.

	Median/*n*	IQR/%
Hospitalisation length, days	5	4–6
Abscess volume, cm^3^	18.01	6.63–42.77
Affected area number	1	1–2
Admission biochemical parameters:		
WBC, ×10^3^/μL	14.25	11.60–17.03
CRP, mg/Ll	138.3	82.5–210.7
Procalcitonin, ng/mL	0.13	0.08–0.38
Main symptoms:		
Pain	94	93.07
Swelling	83	82.18
Trismus	61	60.40
Swallowing difficulties	39	38.61
Fever	22	21.78
Breathing difficulties	7	6.93
Headache	4	3.96
Abscess area:		
Submandibular	67	66.34
Submental	12	11.88
Neck	11	10.89
Pterygomandibular	9	8.91
Submasseteric	9	8.91
Mouth floor	6	5.94
Canine fossa	5	4.95
Perimandibular	4	3.96
Parapharyngeal	3	2.97
Intraoral	2	1.98
Buccal	2	1.98
Preauricular	2	1.98
Subtemporal	2	1.98
Peritonsillar	1	0.99
Maxillary sinus	1	0.99
Orbit	0	0.00
Pterygopalatine	0	0.00
Sublingual	0	0.00
Phlegmon	25	24.75
Empyema	1	0.99
Affected side:		
Maxilla	7	6.93
Mandible	81	80.20
Both/undetermined	13	12.87
Causative tooth:		
Incisor	2	1.98
Canine	1	0.99
Premolar	5	4.95
Molar	30	29.70
Wisdom	48	47.52
Various/undetermined	15	14.85
Anaesthesia:		
General	86	85.15
Local	13	12.87
Reoperation	6	5.94
Antibiotics (prior to):	51	50.50
Amoxicillin + clavulanic acid	10	9.90
Clindamycin	31	30.69
Metronidazole	6	5.94
Others	4	3.96
Antibiotics (hospital):	98	97.03
Amoxicillin + clavulanic acid	32	31.68
Clindamycin	25	24.75
Metronidazole	69	68.32
Cefuroxim	20	19.80
Ceftriaxone	17	16.83
Others	1	0.99

**Table 3:** Comparison of clinical and biochemical parameters across pandemic periods (*p*-values for the Kruskal–Wallis test).

	Pre-Pandemic		Pandemic		Post-Pandemic		*p*-Value
	Median	IQR	Median	IQR	Median	IQR	
Age	39	30–52	33.5	23–39.5	30	25–45	0.265
BMI	24.77	22.31–27.24	24.52	19.44–27.77	23.57	21.60–27.15	0.723
Hospitalisation length, days	5	3–6	4.5	3–8.5	5	4–5	0.883
Abscess volume, cm^3^	22.36	6.63–42.77	10.05	5.55–57.41	17.93	7.50–37.06	0.857
Affected area number	1	1–2	1	1–2	1	1–2	0.982
Admission biochemical parameters:							
WBC, ×10^3^/μL	14.18	12.04–17.91	13.02	9.81–15.79	14.57	11.60–17.03	0.543
CRP, mg/L	122.6	78.5–223.7	134.95	80.2–192.8	143.2	84.1–210.1	0.863
Procalcitonin, ng/mL	0.14	0.07–0.36	0.17	0.09–1.06	0.10	0.05–0.20	0.176

**Table 4:** Detailed parameters of determined points in multidimensional correspondence analysis.

	x	y	Quality	Relative Inertia	x Inertia	x cos^2^	y Inertia	y cos^2^
Period: pre-pandemic	−0.1616	0.5931	0.5686	0.0581	0.0047	0.0188	0.1033	0.2533
Period: pandemic	−0.1836	−1.5861	0.5879	0.0837	0.0023	0.0066	0.2872	0.4892
Period: post-pandemic	0.2330	0.0237	0.6350	0.0581	0.0097	0.0391	0.0002	0.0004
Mandible	−0.2842	0.0267	0.9454	0.0081	0.0317	0.9114	0.0005	0.0080
Maxilla	3.2072	−0.3012	0.9454	0.0919	0.3577	0.9114	0.0052	0.0080
Quadrant: third	−0.2781	−0.3554	0.2372	0.0477	0.0173	0.0849	0.0464	0.1387
Quadrant: fourth	−0.2923	0.5325	0.2421	0.0605	0.0144	0.0559	0.0786	0.1854
Quadrant: first	3.3911	0.5572	0.8861	0.0930	0.3428	0.8625	0.0152	0.0233
Quadrant: second	2.1036	−5.4516	0.6063	0.0988	0.0220	0.0521	0.2424	0.3496
Wisdom	−0.2034	−0.4343	0.5199	0.0442	0.0099	0.0523	0.0738	0.2382
Molar	0.0850	0.3630	0.2207	0.0651	0.0011	0.0039	0.0322	0.0706
Premolar	−0.4874	1.6043	0.2864	0.0942	0.0059	0.0147	0.1050	0.1589
Incisor	2.2779	0.6387	0.1337	0.0977	0.0516	0.1235	0.0067	0.0097
Canine	5.0957	0.6566	0.3696	0.0988	0.1290	0.3055	0.0035	0.0051

**Table 5:** Spearman’s correlation between hospitalisation, abscess volume, and affected area number and demographic and clinical parameters.

	Hospitalisation Length		Abscess Volume		Affected Area Number	
	Rs	*p*-Value	Rs	*p*-Value	Rs	*p*-Value
Age	0.059	0.560	0.118	0.430	0.153	0.127
BMI	0.253	0.011 *	−0.079	0.596	0.144	0.151
Comorbidities	0.195	0.051	−0.050	0.736	0.256	0.010 *
Stimulants	−0.044	0.659	0.055	0.715	0.099	0.325
Hospitalisation length	n/a		0.239	0.106	0.495	<0.001 *
Affected area number	0.495	<0.001 *	0.260	0.078	n/a	
Abscess volume	0.239	0.106	n/a		0.260	0.078
Antibiotics (prior to)	−0.186	0.063	0.188	0.205	−0.119	0.235
Antibiotics (hospital)	−0.222	0.026 *	−0.033	0.828	−0.167	0.096
Admission WBC	0.183	0.066	0.342	0.018 *	0.301	0.002 *
Admission CRP	0.385	<0.001 *	0.104	0.485	0.393	<0.001 *
Admission procalcitonin	0.289	0.005 *	0.046	0.765	0.258	0.013 *

* significant Spearman’s correlation coefficients (Rs).

**Table 6:** Multiple regression modelling for hospitalisation length, abscess volume, affected area number, and reoperation using the stepwise forward selection method.

	Standardised Beta	Standard Error	*p*-Value
Hospitalisation length: R^2^ = 0.815, *p*-value < 0.001			
Intercept			<0.001 *
Admission procalcitonin	0.315	0.098	0.003 *
Admission CRP	0.240	0.088	0.010 *
Abscess volume	0.308	0.090	0.002 *
Reoperation: 1	0.299	0.092	0.003 *
Stimulants: 1	−0.154	0.077	0.054
Antibiotics (prior to): 1	−0.084	0.084	0.323
Abscess volume: R^2^ = 0.480, *p*-value = 0.001			
Intercept			0.142
Admission procalcitonin	0.464	0.130	0.001 *
Affected area number	0.122	0.137	0.381
Period: post-pandemic	−0.274	0.127	0.039 *
Antibiotics (prior to): 1	0.281	0.130	0.038 *
Gender: males	0.224	0.131	0.098
Admission WBC	0.159	0.140	0.264
Affected area number: R^2^ = 0.283, *p*-value = 0.023			
Intercept			0.267
Admission WBC	0.351	0.143	0.019 *
Location: city	0.252	0.143	0.088
Age	0.190	0.141	0.186
Side: mandible	0.161	0.144	0.274
Reoperation: R^2^ = 0.603, *p*-value < 0.001			
Intercept			0.002 *
Hospitalisation length	0.594	0.166	0.001 *
Comorbidities: 1	0.262	0.111	0.025 *
Abscess volume	−0.294	0.137	0.040 *
Admission procalcitonin	0.301	0.163	0.074
Affected area number	0.173	0.113	0.138
Stimulants: 1	0.143	0.110	0.205

* significant predictors for the multiple regression modelling.

## Data Availability

Data are available upon request from the corresponding author.
